# Potential effects of angiogenesis-related factors on the severity of APAC and surgical outcomes of trabeculectomy

**DOI:** 10.1186/s12886-021-02051-w

**Published:** 2021-08-12

**Authors:** Jing Wang, Ming-shui Fu, Min-wen Zhou, Bi-lian Ke, Zhi-hua Zhang, Xun Xu

**Affiliations:** 1 National Clinical Research Center for Eye Diseases, Shanghai, China; 2 Shanghai Key Laboratory of Ocular Fundus Diseases, Shanghai, China; 3 Shanghai Engineering Center for Visual Science and Photomedicine, Shanghai, China; 4 Shanghai Engineering Center for Precise Diagnosis and Treatment of Eye Disease, Shanghai, China; 5 Eye & ENT Hospital, Fudan University, 83 Fenyang Rd, Shanghai, 20000 China; 6 Department of Ophthalmology, Shanghai General Hospital, Shanghai Jiao Tong University, Shanghai, China

**Keywords:** APAC, IOP, EPO, PDGF, trabeculectomy

## Abstract

**Background:**

EPO (erythropoietin) and PDGF (platelet derived growth factor) families are thought to be associated with angiogenesis under hypoxic condition. The sharp rise of intraocular pressure in acute primary angle closure (APAC) results in an inefficient supply of oxygen and nutrients. We aimed to measure the expression of EPO and PDGF family members in APAC eyes and demonstrate their associations with APAC’s surgical success rate.

**Methods:**

Concentrations of EPO, PDGF-AA, -BB, -CC and -DD collected in aqueous humor samples of 55 patients recruited were measured. Before operations, correlations between target proteins and IOP (intraocular pressure) were detected between APAC (acute primary angle closure) and cataract patients. Based on the post-operative follow-up, the effects of EPO and PDGF family members on the successful rate of trabeculectomy were tested.

**Results:**

The levels of EPO, PDGF-CC and -DD were significantly elevated in the APAC group compared to the cataract group. During the post-operative follow-up, EPO, PDGF-CC and -DD showed significant differences between the success and failure groups. In multivariable linear regression analyses, failed filtration surgery was more likely in APAC eyes with higher EPO level. The Kaplan-Meier survival plot suggested that the success rate in eyes with low EPO level was significantly higher than that in eyes with high EPO level.

**Conclusion:**

The levels of EPO, PDGF-CC and -DD were significantly elevated in failure group. EPO level correlated with preoperative IOP and numbers of eyedrops, and higher EPO level in aqueous humor is a risk factor for trabeculectomy failure. It can be a biomarker to estimate the severity of APAC and the success rate of surgery. The investigation of mechanism of EPO in APAC a may have potential clinical applications for the surgical treatment of APAC.

## Introduction

Acute primary angle closure (APAC) is a common ophthalmic emergency. The abrupt closure of trabecular meshwork in the anterior chamber angle causes obstruction of aqueous humor outflow and sudden increase in intraocular pressure, leading to typical symptoms and clinical signs Patients usually refer to emergency management complaining about eye redness, ocular pain or discomfort, blurring of vision, frontal headache, nausea and vomiting. Uncontrollable IOP (intraocular pressure) of eyes leads APAC into angle closure glaucoma after APAC attack. The rate of this altered course of APAC was about 50% under estimation. The sharp rise of IOP in APAC results in an inefficient supply of oxygen and nutrients, which can lead to atrophy of anterior structures, the inflammation of anterior chamber, ischemia and hypoxia of the whole eye [1]. Under reduced oxygen tension, hypoxia inducible transcription factors will be accumulated and lead to over expression of a large variety of target genes such as vascular endothelial growth factor (VEGF), erythropoietin (EPO) and platelet-derived growth factor (PDGF) [2–4].

VEGF is a well-known and potent vasculogenesis and angiogenesis cytokine, which has been found to increase in the aqueous humor of APAC eyes due to hypoxia [5, 6]. Except for VEGF, EPO and PDGF families are also thought to be associated with angiogenesis under hypoxic condition. EPO is a pleiotropic cytokine that was first identified as being essential for red blood cell (RBC) production. It plays a key role in the modulation of the response to injury, inflammation, and tissue hypoxia via the inhibition of apoptosis [7]. The PDGF family includes four homodimers, PDGF-AA, PDGF-BB, PDGF-CC, and PDGF-DD, which are important players in ocular diseases involving the degeneration of retinal neuronal and vascular cells [8, 9]. EPO and PDGFs elevations have been detected in primary open angle glaucoma and neovascular glaucoma [10, 11]. However, there has been no research describing the changes of EPO and PDGF levels in APAC eyes. Recent studies have shown that both EPO and PDGF have neuroprotective functions such as anti-apoptotic [12, 13], anti-oxidant [14] and anti-inflammatory properties [15, 16]. Thus, the fluctuations of their levels in aqueous humor may correlate with prognosis of trabeculectomy in APAC eyes. In this study, We aimed to measure the expression of EPO and PDGF family members (PDGF-AA, PDGF-BB, PDGF-CC and PDGF-DD) in APAC eyes and demonstrate their associations with APAC’s surgical success rate.

## Methods

### Subjects

This study was conducted in accordance with the tenets of declaration of Helsinki, and it was approved by the Institutional Review Board of the Shanghai General Hospital. 55 patients were recruited from Dec 1, 2017 to Mar 31, 2018, prospectively and consecutively. Written and oral informed consent was obtained from each patient and their families, respectively.

A thorough ophthalmic examination including slit-lamp biomicroscopy, IOP measurement by Goldmann applanation tonometry, ultrasound biomicroscopy (UBM), gonioscopy and fundus examinations was made for all the participants. Enrolled APAC patients had experienced an APAC attack within one month before hospital admission. Patients had uncontrolled IOP under standardized antiglaucomatous medications after the pupillary block was relieved using laser peripheral iridotomy. Patients with secondary angle closures, other ocular diseases or histories of previous ocular surgeries were excluded. Cataract patients, who were enrolled as the control group, had no history of IOP higher than 21 mmHg or glaucomatous optic neuropathy. Exclusion criteria were listed as follows: (1) patients with secondary angle closure, such as lens-induced glaucoma, neovascular glaucoma, or uveitis; (2) other ocular diseases, such as diabetic retinopathy, age-related macular degeneration, and pathological myopia; (3) any history of previous ocular surgery.

### Surgical technique and postoperative care

Trabeculectomy combined with cataract surgery was performed by one experienced surgeon for all of the previous APAC patient s[17]. The main surgical steps are described as follows. First, the surgeon made a fornix-based conjunctival flap and dissected a limbus-based 4×4-mm scleral flap. Then a sponge soaked with 5-fluorouracil (25 mg/mL) was placed for 3 minutes underneath the conjunctival flap. After that, the surgical area was washed by 250 mL balanced salt solution. Second, cataract extraction was performed by phacoemulsification and intraocular lens implantation according to the surgeon’s preferred technique. Finally, a trabeculectomy and basal iridectomy were operated. After the procedure, two 10-0 nylon sutured the scleral flap at the corners and two adjustable sutures were made at the centers of the two sides, allowing the minimal leakage during the reconstruction of anterior chamber. Then close the conjunctiva by a 10-0 nylon suture. After surgery, topical antibiotic and topical nonsteroidal anti-inflammatory medication were used four times daily for 1 week. Corticosteroids eye drops were used every 2 to 3 hours for the first week and then reduce the frequency gradually over the next 1 to 2 months. Postoperative interventions of ocular massages followed by loosening the djustable sutures with forceps were performed when IOP >21 mmHg for bleb formation. If the bleb associated with a rise in IOP became encapsulated, paracentesis of anterior chamber was carried out without antimetabolites. To minimize the bias, the type and timing of interventions in post-operative care were also determined by the same surgeon. In the case of postoperative IOP measurements > 21 mmHg, IOP-lowering medication was added with or without an ocular massage or having any adjustable sutures loosened (if needed). In cases of inadequate IOP control, additional surgical procedures were performed as required.

### Aqueous humor collection and analysis

Generally, 80-120μl aqueous humor was needed for an examination and analysis. The 30-gauge needle and tuberculin syringe were used to withdraw the aqueous humor via limbal paracentesis. Aqueous humor samples from APAC or cataract patients were collected at the start of the trabeculectomy or cataract surgery, respectively. The aqueous humor was collected with caution to avoid touching any intraocular tissue. The collection was frozen in liquid nitrogen immediately and transferred within 24 hours into a -80°C environment until the analysis was completed.

EPO and PDGF family members (PDGF-AA, PDGF-BB, PDGF-CC and PDGF-DD) were detected by the Luminex Screening Human Magnetic Assay (R&D Systems, Inc., Minneapolis, MN) according to the manufacturer’s instructions of the Bio-Plex 200 System (Bio-Rad Laboratories, Inc., Hercules, CA). The Bio-Plex Manager 5.1.0.0 software produced the standard curve automatically . The aqueous humor samples were diluted in a 1:2 ratio with calibrator diluent. The standard concentrations on the Certificate of Analysis was used and 3-fold dilutions for the remaining levels were calculate. The duplicate readings for each standard and sample were then averaged. The final levels of the EPO and PDGF family members were analyzed after subtracting the average blank median fluorescence intensity (MFI).

### Outcome measures

The IOPs of all the participants were analyzed a half hour before each surgical procedure, and all procedures were performed during a similar time frame in the afternoon. After operation, all APAC patients were followed up at 1 week and 1, 3, 6, 12 and 18 months. Goldmann tonometry and slit lamp biomicroscopy were performed at each post-operative follow-up. The surgical failure was defined as IOP >21 mmHg or administration of anti-glaucoma medications after 3 months or an additional glaucoma surgery [18, 19]. The ocular massage, loosening of adjustable sutures and bleb needling were performed at the slit lamp and were not considered as an additional glaucoma surgery. If the IOP of one visit was lower than 21 mmHg without the use of topical anti-glaucoma medication, the visit was defined as a “success”.

### Statistical analysis

Commercially available SPSS software (version 26.0.0.0, SPSS Inc., Chicago, IL, USA) was used to analyze the results. The Kolmogorov-Smirnov test was used to test the normality of the continuous variable. The median (M) value and interquartile range (IQR) were used to describe the distribution of EPO, PDGF-AA, PDGF-BB, PDGF-CC and PDGF-DD. A Mann-Whitney U test was used to perform the comparisons of target proteins and the clinical characteristics between the two groups. A Spearman correlation test was performed to determine the correlation between target proteins and associated factors.

After operation, a Mann-Whitney U test was used to perform the comparisons of EPO and PDGF families between success and failure groups. If any target protein was detected to have a significant difference between the two groups, it was included in the multiple linear regressions to characterize the effects after adjustment. If any protein showed significance after multiple linear regressions, the patients would be divided into two groups according to the protein level. The Kaplan Meyer survival curves were used to display the success rate of the end points over time between the low and high protein groups. Also, in the failure group, a Spearman test was used to examine the correlation between significant-protein(s) and the IOP and the time of failure before the use of any drug or surgical treatment. A p-value less than 0.05 was regarded as being statistically significant.

## Results

### Patients

In total, 55 patients participated in the present study, of which 35 were previous APAC patients and 20 were non-glaucomatous cataract patients. The clinical characteristics of patients are shown in Table 1. Since each data group was non-normally distributed, we used M and IQR values to present the data distribution. There were no significant differences between the APAC group and the control group regarding age (p=0.630) or sex (p=0.138). The IOP of these two groups had a significant difference (p<0.001).

**Table 1:** Clinical characteristics of the study patients

Characteristics	Previous APAC	Cataract	P
No. Patients (No. Eyes)	35 (35)	20 (20)	/
Age, Medium (IQR), years	70 (14)	67 (20)	0.630
Sex [n (%)]			0.138
Male	12 (34.3%)	11 (55.0%)	
Female	23 (65.7%)	9(45.0%)	
IOP ,Medium (IQR), mmHg	31.0(13.0)	14.0 (2.0)	<0.001*

Note:Mann-Whitney U test test was used to investigate the difference of age, gender and IOP between two groups. *P<0.05

*APAC* acute primary angle closure, *No*. number, *IOP* intraocular pressure, *IQR* interquartile-range

### EPO and PDGF

The EPO and four PDGF family members were investigated in the aqueous humor samples of all 55 patients. It was found that the EPO and two PDGF family members, PDGF-CC and PDGF-DD, had significant differences between the APAC and the control group (Table 2). The Spearman correlation analysis between the levels of five target proteins and age, sex, IOP, number of preoperative antiglaucoma eyedrops as well as interval between the onset of APAC and trabeculectomy is shown in Table 3. In the previous APAC group, EPO had a significant correlation with pre-operation IOP and number of preoperative eyedrops, PDGF-AA and PDGF-BB only had a significant correlation with pre-operation IOP, while PDGF-DD had a significant correlation with sex and interval between the onset of APAC and trabeculectomy.

**Table 2:** Aqueous humor analysis of EPO and PDGF family members concentration in two groups

Analyte	APAC	Cataract	Median difference (95% CI)	P
EPO, Medium (IQR), pg/ml	36.46 (16.74)	25.96 (10.50)	9.00 (0.00-10.50)	0.009*
PDGF-AA, Medium (IQR), pg/ml	1.92 (0.74)	1.62 (0.78)	0.18 (-0.16-0.54)	0.302
PDGF-BB, Medium (IQR), pg/ml	0.18 (0.04)	0.16 (0.02)	0.00 (0.00-0.20)	0.135
PDGF-CC, Medium (IQR), pg/ml	1.90 (2.02)	1.44 (0.51)	0.40 (0.14-0.72)	0.002*
PDGF-DD, Medium (IQR), pg/ml	0.10 (0.12)	0.04 (0.00)	0.04 (0.02-0.12)	<0.001*

Note: Mann-Whitney U test was used to investigate the difference of EPO, PDGF-AA, PDGF-BB, PDGF-CC and PDGF-DD between APAC and cataract patients’ aqueous humor. *P<0.05

*APAC* acute primary angle closure, IQR= interquartile-range

**Table 3:** Correlations between target proteins and associated factors in two groups

Spearman Correlation		Previous APAC				
Target Protein (pg/ml)		Age	Gender	Preoperative IOP	Number of preoperative eyedrops	Interval between the onset of APAC and trabeculectomy
EPO	r	-0.177	-0.137	0.343*	0.404*	-0.048
	P	0.309	0.433	0.044	0.016	0.786
PDGF-AA	r	0.132	-0.101	0.444*	0.225	-0.230
	P	0.449	0.562	0.008	0.193	0.183
PDGF-BB	r	-0.125	-0.142	0.515*	0.293	-0.324
	P	0.473	0.417	0.002	0.087	0.058
PDGF-CC	r	0.152	-0.153	0.317	0.115	-0.217
	P	0.384	0.382	0.064	0.512	0.211
PDGF-DD	r	0.029	-0.348*	0.137	0.086	-0.454*
	P	0.876	0.040	0.432	0.624	0.006

Note:Spearman correlation test was used to evaluate the correlations between EPO, PDGF-AA, PDGF-BB, PDGF-CC and PDGF-DD and demographic characteristic in previous APAC group. *P<0.05

*APAC* acute primary angle closure, *IOP* intraocular pressure

### Surgical results

After operation, all of the APAC patients finished their follow-ups after 18 months. During the follow-up period, 24 eyes of the 35 previous APAC eyes (68.6%) were assigned to the success group, and 11 eyes (31.4%) were assigned to the failure group. The age, gender, numbers of preoperative eyedrops, the interval between the onset of APAC and trabeculectomy and pre-operation IOP showed no significant differences between the two groups (Table 4).

**Table 4:** Postoperative clinical characteristics of the success and failure group

Characteristics	Success group	Failure group	P
No. Patients (%)	24 (68.6%)	11 (31.4%)	/
Age, Medium (IQR), years	72(13.5)	65(18.0)	0.061
Sex [n (%)]			0.283
Male	10 (41.7%)	2 (18.2%)	
Female	14 (58.3%)	9(81.8%)	
IOP ,Medium (IQR), mmHg	34.5 (16.3)	40.0 (21.0)	0.123
No. of Preoperative Eyedrops, Medium (IQR)	2 (1.8)	2 (2.0)	0.985
Interval between the Onset of APAC and Trabeculectomy, Medium (IQR), days	7.75 (14.0)	9 (7.0)	0.113

Note: Mann-Whitney U test test was used to investigate the difference of age, gender, IOP, numbers of preoperative eyedrops, and interval between the onset of APAC and trabeculectomy between two groups

*APAC* acute primary angle closure, *No*. number, *IOP* intraocular pressure, *IQR* interquartile-range

**Table 5:** Aqueous humor analysis of EPO and PDGF family members concentration in success and failure groups

Analyte	Success	Failure	Median difference (95% CI)	P
EPO, Medium (IQR), pg/ml	28.44 (10.50)	48.60 (40.68)	-16.74 (-37.22~-6.24)	0.008*
PDGF-AA, Medium (IQR), pg/ml	1.62 (0.71)	2.14 (1.18)	-0.50 (-1.06~0.04)	0.074
PDGF-BB, Medium (IQR), pg/ml	0.16 (0.04)	0.18 (0.12)	-0.02 (-0.06~0.00)	0.164
PDGF-CC, Medium (IQR), pg/ml	1.70 (0.42)	2.46 (7.14)	-0.72 (-2.32~-0.30)	0.003*
PDGF-DD, Medium (IQR), pg/ml	0.08 (0.12)	0.18 (0.28)	-0.060 (-0.20~0.00)	0.045*

Note: Mann-Whitney U test was used to investigate the difference of EPO, PDGF-AA, PDGF-BB, PDGF-CC and PDGF-DD between success and failure patients’ aqueous humor. *P<0.05

*APAC* acute primary angle closure, *IQR* interquartile-range

Three eyes (12.5%) in the success group and two eyes (18.1%) in the failure group received ocular massages. Five eyes (20.8%) in the success group and five eyes (45.5%) in the failure group received suture lysis. The differences were not significant. There was a slightly greater number of eyes receiving needling in the failure group (4/11 eyes) than in the success group (3/24 eyes), but the difference was not significant (p=0.1013). In the failure group, two patients underwent glaucoma valve implantation, and two underwent ciliary body photocoagulation; two needed two kinds of intraocular pressure-lowering medication to control their IOP, and five needed one kind. All post-operation patients who used IOP-lowering medications had their IOP controlled within the normal range.

### Correlation between EPO/PDGFs and surgical outcomes

**Fig. 1:**
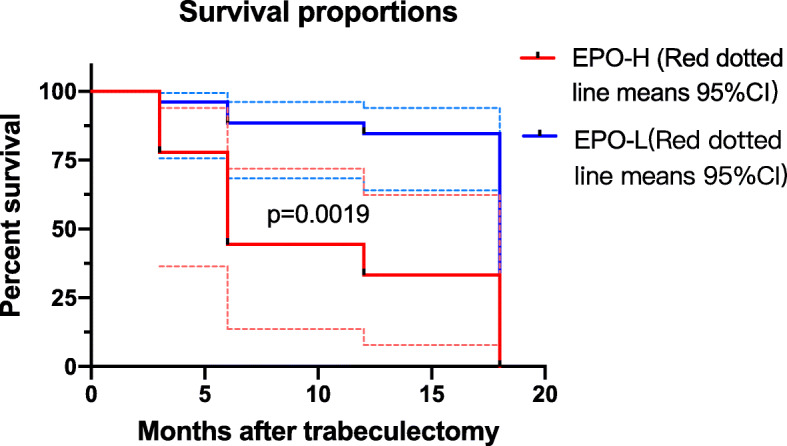
Surgical Results Between Groups With Low and High Levels of EPO. Kaplan-Meier survival plot of two groups according to their aqueous humor EPO levels: eyes with EPO value < 36.46 ng/mL (group L, 17 eyes, solid line) and eyes with SPARC value > 36.46 ng/mL (group H, 18 eyes, dashed line). The probability of success of trabeculectomy for eyes with low EPO levels was significantly higher than that for eyes with high EPO levels based on the through Breslow (Generalized Wilcoxon) test (P = 0.003)

We first determined the potential changes in EPO and PDGF levels between the success and failure groups. Our results showed that the EPO, PDGF-CC and PDGF-DD levels were significantly elevated in the failure group (Table 5). Then the age and content of EPO, PDGF-CC, and PDGF-DD were included in the multiple linear regression analyses (Table 6). Failed filtration surgery was more likely in APAC eyes with higher EPO level (p=0.021). Other factors, including age and the levels of PDGF-CC and PDGF-DD, were not associated with the outcome of trabeculectomy. Results indicated that high EPO level was a significant risk factor for trabeculectomy failure. We, subsequently, divided the patients into two groups according to the levels of these two proteins, respectively. To EPO, 17 eyes with EPO levels lower than the median value (<36.46 ng/mL) were included in group EPO-L, and the other 18 eyes were divided in group EPO-H. The Kaplan-Meier survival plots were drawn to investigate the success rate between the EPO-L and EPO-H groups (Fig. 1). It showed that patients with low EPO levels had significantly higher success rates over the 18-month follow-up period compared to those with high EPO levels: with p=0.003 through Breslow (Generalized Wilcoxon) test.

**Table 6:** Multivariate analyses of the associations between age, EPO, PDGF-CC, PDGF-DD and the outcome of surgery

Factors	Standardized coefficient	Unstandardized coefficient	p
Age	0.269	0.013	0.086
EPO, pg/ml	-0.371	-0.003	0.021*
PDGF-CC, pg/ml	-0.112	-0.005	0.577
PDGF-DD, pg/ml	-0.280	-0.280	0.086

Note:Multivariable linear regression analyses were performed with outcome of surgery as the dependent variables and age, EPO, PDGF-CC and PDGF-DD as independent variables. *P<0.05

## Discussion

APAC is an ophthalmic emergency characterized by the sharp rise of IOP and ischemia of the eye. Aqueous humor contains various protein factors, and the composition of those proteins changes dramatically with IOP elevation and ischemia [20]. EPO is a hematopoietic glycoprotein playing an important role in the modulation of the response to tissue hypoxia via the inhibition of apoptosis [7, 21]. In a biological study, EPO proved to be effective in preventing the loss of retinal ganglion cells (RGCs) due to pressure elevation [22]. Researches also showed that PDGFs play critical roles in many ocular neovascular diseases and RGCs as well as degeneration diseases, such as glaucoma, retinopathy of prematurity and retinitis pigmentosa [23, 24]. Recently, the PDGF-AA protein has been shown to rescue RGCs from death in a glaucoma mice model [25]. Moreover, PDGF-CC has proved to be critically required for neuronal survival in an ischemia-induced stroke model [26]. Thus, the levels of EPO and PDGFs could change to cope with the IOP fluctuation induced hypoxia.

In the present study, we found that EPO and two members of the PDGF families (PDGF-CC and -DD) showed significant elevation in the APAC group than in the control group. In other clinical researches, EPO and PDGF were found to increase in the aqueous humor of patients with primary angle closure glaucoma, primary open angle glaucoma and neovascular glaucoma [10, 27]. Mokbel et al pointed out that EPO level increased in aqueous of these glaucoma patients as a result of glaucoma damage and not a cause [28]. In our study, when the APAC occurred, the sudden elevation of IOP caused transient retinal hypoxia. The anoxic environment induced an increase in EPO and PDGF-CC/-DD levels in the eye mainly to serve their neuroprotective functions [15, 29, 30]. Moreover, the EPO level was found to significantly correlate with pre-operation IOP and numbers of preoperative eyedrops. Taken together, it is reasonable to postulate that the EPO level might reflect the severity of the disease in APAC eyes. In severe cases with higher IOP and more usage of medications, EPO was consistently produced to protect retinal cells in a more severe oxygen-deficient environment.

In addition to the neuro- and tissue-protection abilities, EPO and PDGF were proven to associate with the fibrosis and inflammation process in different organs [15, 16, 31–33]. Thus, the fluctuation of these proteins could correlate with the outcome of trabeculectomy. Bearing this in mind, we first detected the EPO and PDGFs levels in the surgical failure group and in the success group, and the content of EPO, PDGF-CC and PDGF-DD showed a significant elevation in the failure group. To further evaluate which proteins were the most relevant risk factors for surgical failure, we performed multiple linear regressions analyses. The result revealed that the level of EPO was significant risk factor for trabeculectomy failure. The Kaplan-Meier survival plots also confirmed that a lower probability of success was achieved in the APAC group with a higher EPO level. Since EPO significantly correlated with preoperative IOP and numbers of antiglaucoma medications usage, it is possible that eyes with higher preoperative IOP and more usage of eyedrops had worse conditions. In those conditions, retinal ganglion cells called for much higher level of EPO to fight against the oxygen-deficient environment. Some studies have shown the anti-fibrosis [33] and anti-inflammation [16] abilities of EPO. However, various factors in the aqueous humor were found rising during acute angle closure, such as matricellular proteins and inflammation factors. The former can cause the extracellular deposition of fibrillary and obstruct the outflow route [34, 35], and the latter can cause the function loss of the blebs [5]. Therefore, we speculated that even though EPO was more elevated to protect the RGCs in severe cases, the level of EPO was not high enough to completely reverse the fibrosis, and it ultimately resulted in surgical failure. Researches should further analysis the correlations between EPO and other factors affecting the outcome of trabeculectomy.

The study is translational to clinical practice in that it reminds us that EPO therapy is a double-edged sword in the treatment of eye disease. Different modes of EPO administration, such as systemic delivery or intravitreal injection, have given positive results for ocular disorders [2, 36, 37]. There is emerging evidence for the neuroprotection effect of EPO both in vivo and in vitro [21, 38, 39]. The positive roles that EPO may play in APAC under higher dose, such as anti-apoptotic [12, 13], anti-oxidant [14], and anti-inflammatory [15, 16] make it an ideal treatment in APAC. However, its role in angiogenesis, causing neovascularization is a dreaded occurrence in the eye [40]. Future animal studies should be performed to verify our theory and, hopefully, get the optimal dosage and regime of EPO treatment after APAC.

There were several limitations in our current study. First, although we performed postoperative follow-ups for patients, due to ethical limitations, we were unable to detect the EPO and PDGF levels in their aqueous humor after surgery. Therefore, we cannot conclude a causal relationship between the EPO and the surgical outcomes, and future animal studies are needed to verify our results by dynamic observation. Second, the sample size of this study was too small to perform a subgroup analysis, and the number of subjects in each group was slightly uneven (although the differences in age and sex between the groups were not significant). Third, the interval between the onset of APAC and trabeculectomy did not show any association with the surgical outcomes (p=0.275 for univariate analysis and p=0.243 for multi-factor regression analysis). The lack of association with surgery time after APAC is surprising, because it seems earlier surgery performed can achieve better results in clinical working. Future large number trials should be performed to verify this. Future research should also be performed to check if there is any difference in the EPO and PDGF levels between the different types of failures, and to check if there is any causal relationship between the levels of EPO and the number of pre-operative medications usage. However, to our knowledge, this is the first study evaluating the levels of EPO and PDGF family members in APAC eyes. A higher successful rate of trabeculectomy was found in the low-EPO level group.

## Conclusions

The levels of EPO, PDGF-CC and PDGF-DD were significantly elevated in eyes suffering from APAC. Eyes with higher EPO levels had lower successful rates of trabeculectomy, so the EPO content in aqueous humor may be used as a predictor of APAC prognoses. Further studies are necessary to investigate the molecular mechanism(s) and histological evidence of EPO pathogenesis in APAC and targeting its expression may have potential clinical applications for the surgical treatment of APAC.

## Data Availability

The datasets used and/or analysed during the current study are available from the corresponding author on reasonable request.

## Abbreviations

EPO: Erythropoietin
PDGF: Platelet derived growth factor
APAC: Acute primary angle closure
IOP: Intraocular pressure
VEGF: Vascular endothelial growth factor
RBC: Red blood cell
UBM: Ultrasound biomicroscopy
MFI: Median fluorescence intensity
M: Median
IQR: Interquartile range
EPO-L: EPO levels lower than the median value
EPO-H: EPO levels higher than the median value
RGCs: Retinal ganglion cells
